# Profiling heterogenous sizes of circulating tumor microemboli to track therapeutic resistance and prognosis in advanced gastric cancer

**DOI:** 10.1007/s13577-021-00568-2

**Published:** 2021-06-21

**Authors:** Yang Chen, Jiajia Yuan, Yanyan Li, Xue Li, Ying Yang, Jian Li, Yilin Li, Lin Shen

**Affiliations:** Department of Gastrointestinal Oncology, Key Laboratory of Carcinogenesis and Translational Research (Ministry of Education), Peking University Cancer Hospital and Institute, Fucheng Road 52, Haidian District, Beijing, 100142 China

**Keywords:** Gastric cancer, CTM, CTC, Resistance, Prognosis

## Abstract

Circulating tumor microemboli (CTM) aggregated by ≥ 2 circulating tumor cells (CTCs) are more migratory than single CTCs. Aside from the plasticity in their molecular characteristics, which have been considered tumor migration, CTM also possesses high size heterogeneity. This study, therefore, systematically investigated the heterogeneous sizes of CTM and their involvement in therapeutic resistance in 114 patients with advanced gastric cancer (GC) using a pre-established surface molecule-independent subtraction enrichment (SE)-iFISH strategy. CTM, which was pre-therapeutically detected in 33.3% of GC patients, can further form in another 34.78% of patients following chemo-/targeted therapies. The presence of CTM is relevant to liver metastasis as well as higher CTC levels (≥ 5/6 mL). Further size-based profiling of GC-CTM revealed that CTM with 2 CTCs (CTM_2_) was the dominant subtype, accounting for 50.0% of all detected GC-CTMs. However, CTM with 3–4 CTCs (CTM_3–4_) specifically associates with chemo-/targeted therapeutic resistance and inferior prognosis. Patients with ≥ 1 CTM_3–4_/6 mL have shorter median progression-free survival and median overall survival. Unlike CTM_2_ and CTM_3–4_, which are detectable in pre-therapy and post-therapy, larger aggregated CTM_≥5_ (CTM with ≥ 5 CTCs) was only intra-therapeutically detected in four HER2^+^ GC patients, of which three experienced liver metastases. Obtained results suggested that the cluster size of GC-CTM should be dynamically profiled beyond pre-therapeutic whole CTM enumeration in terms of chemo-/targeted resistance or metastasis monitoring. GC-CTM_3–4_ could be a potential indicator of therapeutic resistance, while the dynamic presence of GC-CTM_≥5_ implies liver metastasis in HER2^+^ GC patients.

## Introduction

Circulating tumor microemboli (CTM) are clusters of two or more circulating tumor cells (CTCs), which always co-exist with isolated CTCs in peripheral blood during tumor dissemination and metastasis [1]. Although CTM is a minority in the overall CTC population, it has been demonstrated to be more responsible for distant malignant colonization and tumor recurrence compared to single CTC [1–4]. It is quite clear that the heterogeneous clustering of polyclonal cells contributes to the metastatic advantages of CTM [5–8]. For homotypic CTMs made of only CTCs, the cellular heterogeneity of clustered CTCs (such as undifferentiated vs. differentiated and epithelial vs. EMT) could provide a competitive advantage for colonization at distant sites. For heterotypic CTMs made of CTCs and other stromal/immune cells), the cooperativity and crosstalk between diverse cells may facilitate immune escape and prompt CTM survival and proliferation [3].

Different numbers of aggregated cells also confer a highly heterogeneous cluster size to CTM [4, 9–11]. However, how the heterogeneous sizes of CTMs affect their metastatic capacity remains controversial. Based on the assumption that CTMs with larger sizes could have lower velocities, some studies speculate that larger CTMs are much easier to be intercepted by small vessels and seed metastatic tumors than smaller ones [12]. Paradoxically, other studies argued that larger CTMs are more difficult to transit through capillaries than smaller CTMs, which consequently prevents them from colonizing in distant organs [13, 14]. A probable explanation for this contradiction could be the plastic size and morphology of the CTM. An in vitro study on the physical behaviors of breast cancer cell lines showed that cell clusters containing ≥ 20 cells could traverse capillaries by automatic dissociation into individual cells, which can be substantially reorganized to promote their resistance to fluid shear stress [15]. Even so, observations from clinical studies suggest that CTM aggregated by 2 − 5 cells, rather than the larger ones, were dominant in breast cancer patients [10], implying much more intricate aggregate behaviors of CTM during their transportation and dissemination.

Few studies in gastric cancer (GC) have so far focused on the potential prognostic disparities of CTM with different cluster sizes, although the presence of CTM has also been demonstrated to be inversely associated with overall survival (OS) in GC [16, 17]. Furthermore, our recent studies demonstrated that heterogeneous-sized GC CTCs harbor distinct genetic signatures and, in turn, proceed chemo-/targeted therapeutic resistance via diverse mechanisms [18], which raises another paralleling question of whether heterogeneous-sized CTM also differentially contributes to therapeutic resistance and tumor recurrence.

In the present study, taking advantage of the pre-established surface molecule-independent subtraction enrichment (SE)-iFISH strategy [19–21], the heterogeneously aggregated sizes of GC-CTM and their impacts on chemo-/targeted therapeutic resistance in GC were studied based on our previously reported GC CTC clinical cohort [21]. In particular, the longitudinal variations of cluster sizes of CTM were investigated in this study to unravel the specific aggregated pattern of GC-CTM involved in chemo-/targeted therapeutic resistance.

## Materials and methods

### Patient enrollment and specimen collection

This cohort was first described in a previous study [21]. A total of 114 patients with advanced GC were enrolled at the Peking University Cancer Hospital from January 2015 to February 2017. All patients (≥ 18 years old) with Karnofsky performance status (KPS) ≥ 70 had locally advanced, recurrent, and/or histopathologically confirmed metastatic adenocarcinoma at either the stomach or gastroesophageal junction. Patients were subjected to first-line paclitaxel or cisplatin-based chemotherapy with or without trastuzumab based on the histopathological HER2 status.

Clinical responses were evaluated once every 6 weeks by computed tomography scanning according to the Response Evaluation Criteria in Solid Tumors (RECIST, version 1.1). Responses were categorized as stable disease (SD), partial response (PR), or progressive disease (PD). Censoring occurred if the patients were still alive at the last follow-up.

Six milliliters (mL) of blood was periodically collected from all recruited 114 patients at baseline. Among 114 subjects, 103 underwent longitudinal CTC and CTM assessment performed immediately before the beginning of each treatment cycle, and the remaining eight patients were not available for the scheduled post-therapeutic assessments due to unforeseeable clinical complications.

This study was approved by the Ethics Review Committee of Peking University Cancer Hospital, Beijing, China. Written consent forms were obtained from each patient before blood collection. The clinical study was performed following the principles of the Declaration of Helsinki.

### CTM detection using SE-iFISH

The experiment was performed following the manufacturer’s protocol (Cytelligen, San Diego, CA, USA) [21]. Briefly, 6 mL of blood was centrifuged to separate the plasma. Sedimented blood cells were resuspended in 3 mL hCTC buffer and subsequently loaded on top of the non-hematologic cell separation matrix. Samples were centrifuged, followed by collecting the entire solution above red blood cells (RBCs). The solution containing the WBCs was incubated with magnetic beads conjugated to anti-WBC mAbs. WBC-bound immuno-beads were subsequently removed using a magnetic stand. The remaining non-hematologic cells were mixed with the cell fixative, smeared on the formatted CTC slides, and dried for subsequent iFISH processing.

Dried monolayer cells on the coated CTC slides were hybridized with a centromere probe 8 (CEP8) Spectrum Orange (Vysis, Abbott Laboratories, Chicago, IL, USA). Samples were subsequently incubated with an anti-CD45 monoclonal antibody conjugated to Alexa Fluor (AF) 594. After washing, the samples were mounted with mounting media and subjected to the automated Metafer-i·FISH^®^ CTC 3D scanning and image analysis system co-developed by Carl Zeiss (Oberkochen, Germany), MetaSystems (Altlussheim, Germany), and Cytelligen [22]. These CD45^−^ cells with amplified chromosome 8 were identified as CTCs, and the cell clusters consisting of ≥ 2 CTCs were recognized as CTM.

### Statistical analysis

All statistical analyses were performed using SPSS software (version 21.0; IBM Corp., Armonk, NY, USA). The correlations of CTM numbers with clinicopathologic characteristics and clinical responses were assessed using the Pearson *w*^2^-test and Fisher’s exact test, respectively. Progression-free survival (PFS) was defined as the time from initial treatment to the date that clinical progression was confirmed or censored at the last follow-up. Overall survival (OS) was defined as the time from the initial treatment to the date of death or censored at the last follow-up. Kaplan–Meier survival plots for PFS or OS were generated based on the number of CTMs. All *P* values were two-sided, and a *P *value of less than 0.05 was considered statistically significant.

## Results

### The prevalence of CTM and their clinicopathological associations in advanced GC (AGC) patients

**Fig. 1:**
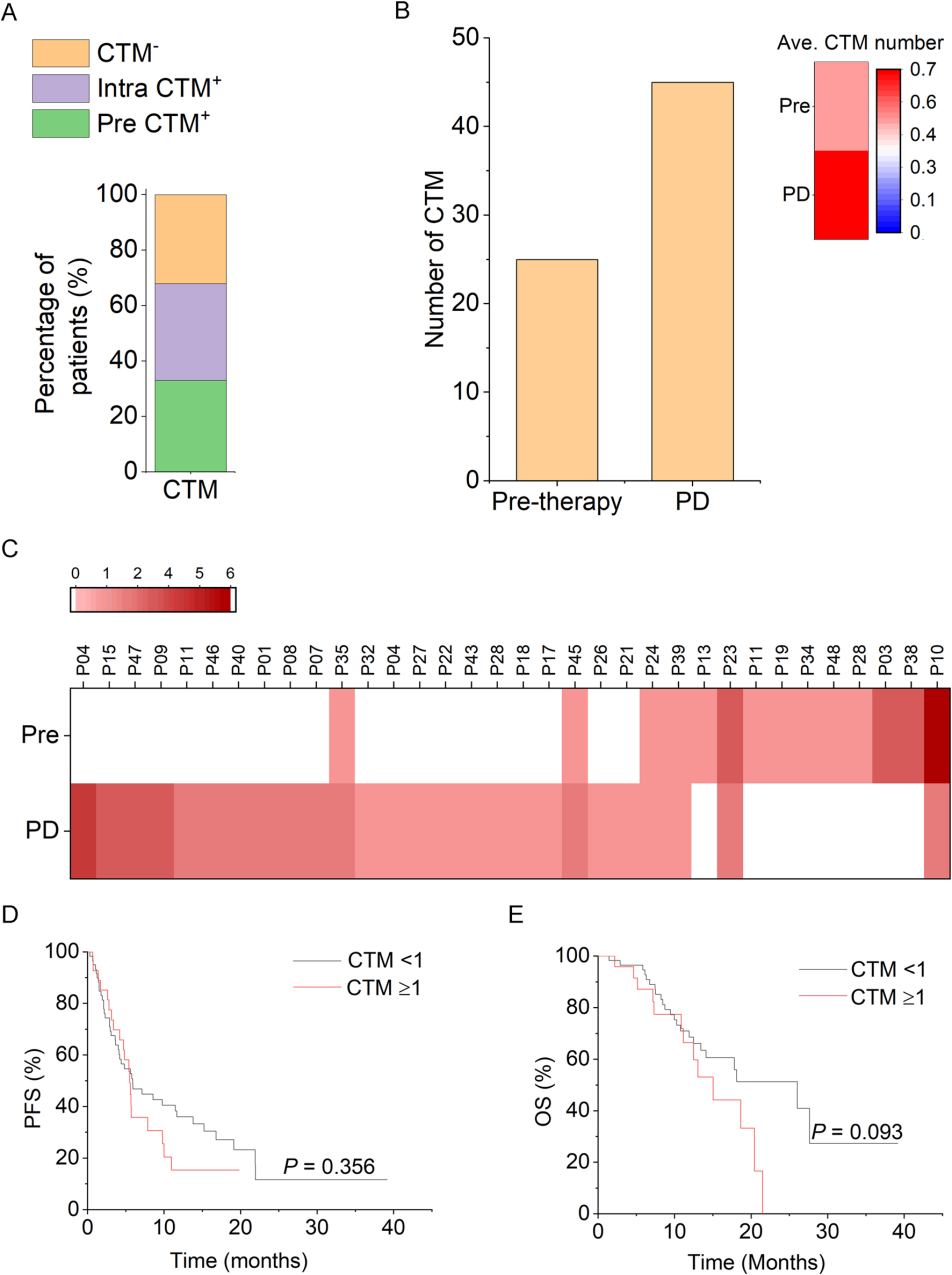
Quantitative variation of GC-CTM following treatment and its relevance to prognosis. **A** Percentages of patients with pre-therapeutic, acquired intra-therapeutic CTM^+^ and CTM^−^. **B** Quantitative comparison of CTM number before treatment and post-PD. The histograms and heatmaps, respectively, indicate the total and average number of CTM before treatment and post-PD. **C** A heatmap shows quantitative variations in CTM before treatment and post-PD in individual PD patients. Increased or decreased CTM numbers are indicated by red or blue color in the heatmap, the white color represents no detectable CTM. **D**, **E** Kaplan–Meier curves of PFS (**D**) and OS (**E**) in relation to pre-therapeutically CTM enumeration

This study was based on our previously reported GC CTC clinical cohort, in which 114 patients with AGC were enrolled [21]. Pre-therapeutic CTM (≥ 1) was detected in 33.3% (38/114) of the patients (Table 1 and Fig. 1A). Specifically, the CTM positivity rate was significantly higher in patients who had liver metastasis or had higher CTC levels (≥ 5/6 mL), indicating that the formation of CTM, which positively correlates with the elevation of CTC number in peripheral blood, can potentially fuel liver metastasis in AGC.

**Table 1:** The associations of pre-therapeutic CTM and clinicopathological characteristics in AGC patients

Variable	All patients	CTM-negative subjects	CTM-positive subjects^1^	*P* Value
Number	114	76 (66.7%)	38 (33.3%)	–
Age, years				
< 60	46	31 (67.4%)	15 (32.6%)	0.529
≥ 60	68	45 (66.2%)	23 (33.8%)	
Gender				
Male	93	63 (67.7%)	30 (32.3%)	0.393
Female	21	13 (61.9%)	8 (38.1%)	
Primary tumor site				
Non-EGJ	66	45 (68.2%)	21 (31.8%)	0.419
EGJ	48	31 (64.6%)	17 (35.4%)	
Lauren classification				
Intestinal	67	47 (70.1%)	20 (29.9%)	0.454
Diffused	15	8 (53.3%)	7 (46.7%)	
Mixed	22	13 (59.1%)	9 (40.9%)	
Liver metastasis				
Yes	59	34 (57.6%)	25 (42.4%)	**0.027**
No	55	42 (76.4%)	13 (23.6%)	
Peritoneum metastasis				
Yes	17	12 (70.6%)	5 (29.4%)	0.472
No	97	64 (66.0%)	33 (34.0%)	
Bone metastasis				
Yes	12	8 (66.7%)	4 (33.3%)	0.616
No	102	68 (66.7%)	34 (33.3%)	
Lung metastasis				
Yes	18	12 (66.7%)	6 (33.3%)	0.599
No	96	64 (66.7%)	32 (33.3%)	
Lymph node metastasis				
Yes	92	60 (65.2%)	32 (34.8%)	0.618
No	22	16 (72.7%)	6 (27.3%)	
CTC number				
27.3 ≥ 5/6 mL	78	45 (57.7%)	33 (42.3%)	**0.002**
< 5/6 mL	36	31 (86.1%)	5 (13.8%)	

^1^≥ 1 CTM/6 mL is identified as CTM-positive

### Dynamic formation of GC-CTM following treatments and its correlation to resistance

Longitudinal detection of GC-CTM following chemo-/targeted treatment showed that another 34.78% of patients who were pre-therapeutic CTM-negative were detected as CTM-positive during their treatment (Fig. 1A), suggesting that CTM can be continually formed during treatment. Further insight into the relationship between the post-therapeutic presence of CTM and resistance in 34 patients who suffered PD at the time of analysis and had positive CTM enumeration either before treatment or after treatment showed that both total and average CTM number surged at the time of PD (Fig. 1B), which was further supported by the individuals’ progression heatmap showing that 64.7% (22/34) of patients experienced CTM elevation when PD was developed (Fig. 1C). Nevertheless, the pre-therapeutic CTM number was not observed to be related to PFS and OS in our study (Fig. 1D, E).

### CTM_*2*_ is the dominant subtype in GC-CTM, while CTM_3–4_ mainly involves in therapeutic resistance

**Fig. 2:**
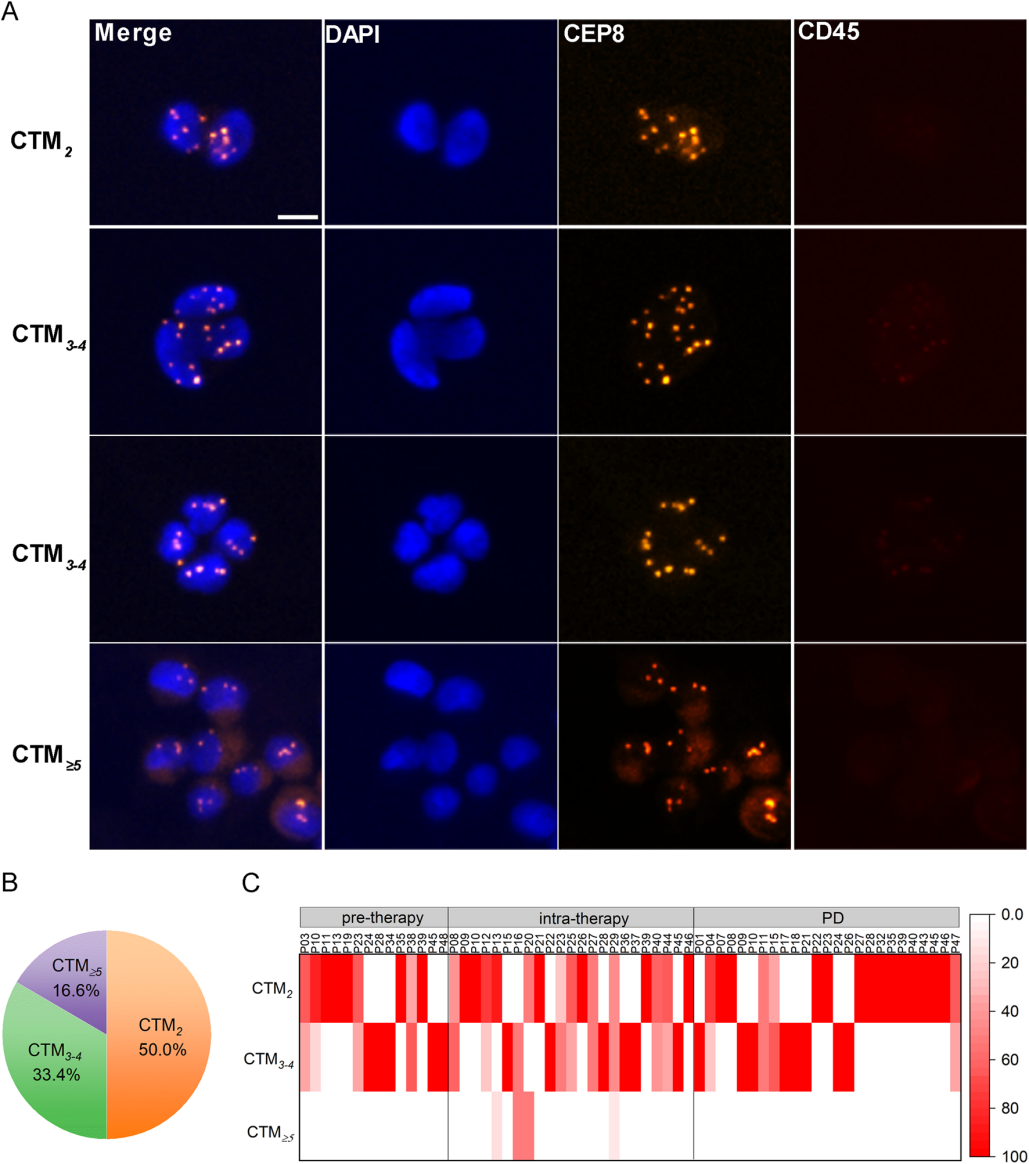
Heterogeneous cluster sizes of GC-CTM and their prevalence following treatments. **A** Typical images of CTM with different aggregated sizes. Bar, 5 μm. **B** Pie chart represents the proportions of CTM_2_, CTM_3–4,_ and CTM_≥5_ in all detected CTM. **C** Heatmap shows the enumeration of CTM_2_, CTM_3–4,_ and CTM_≥5,_ respectively, pre-treatment, intra-treatment, and post-PD in the individual patient

We further questioned whether CTMs with distinct cluster sizes contribute differently to the development of therapeutic resistance. As shown in Fig. 2A, GC-CTM aggregated by 2 CTCs (CTM_2_), 3–4 CTCs (CTM_3–4_), or ≥ 5 CTCs (CTM_≥5_) can all be found, while their percentages in detected GC-CTM are disparate. As shown in Fig. 2B, CTM_2_ was the dominant subtype, accounting for 50.0% of all detected GC-CTMs, followed by CTM_3–4_ (33.4%) and CTM_≥5_ (16.6%).

**Fig. 3:**
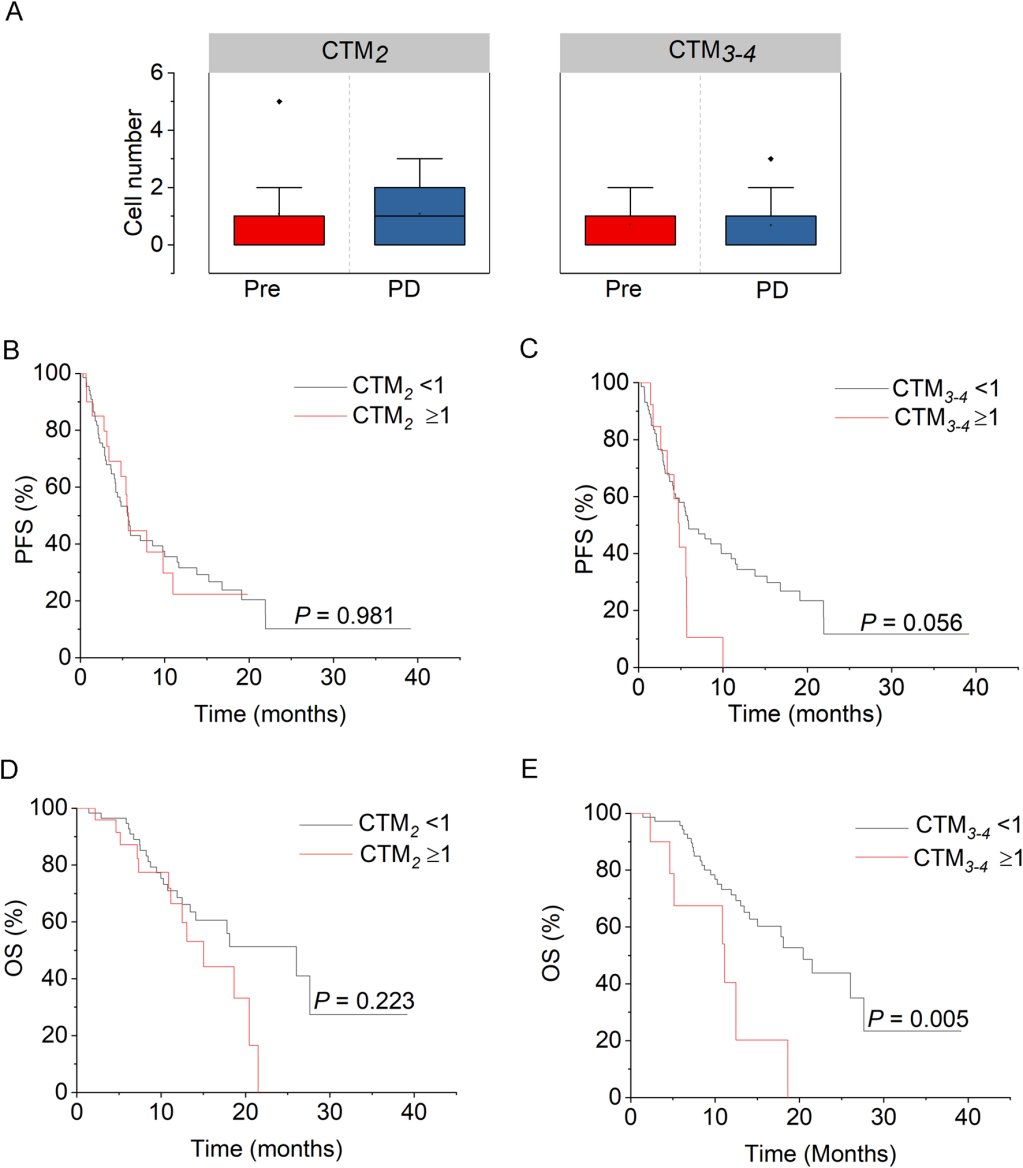
The associations of CTM_2_ or CTM_3–4_ enumeration with therapeutic resistance and prognosis. **A** The boxed chart shows the distributions of CTM_2_ and CTM_3–4_ numbers before treatment and post-PD. **B**, **C** Kaplan–Meier curves of PFS in relation to the pre-therapeutic number of CTM_2_ (**B**) and CTM_3–4_ (**C**). **D**, **E** Kaplan–Meier curves of OS in relation to the pre-therapeutic number of CTM_2_ (**D**) and CTM_3–4_ (**E**)

Dynamically quantitative variations of CTM_2_, CTM_3–4_, and CTM_≥5_ following treatments are also heterogeneous. Unlike CTM_2_ and CTM_3–4_, which were always detectable following the treatments, CTM_≥5_ failed to detect both before treatment and at the time of PD (Fig. 2C). Only intra-therapeutic presence of CTM_≥5_ was observed in four patients with AGC (Fig. 2C). Meanwhile, although CTM_2_ and CTM_3–4_ both exhibited no significant increase when PD was developed (Fig. 3A), the pre-therapeutic CTM_3–4_ was found to be associated with inferior PFS and OS in Kaplan–Meier analysis (Fig. 3B–E). As shown in Fig. 3C, the median PFS (mPFS) of patients with positive CTM_3–4_ level (≥ 1 CTM_3–4_/6 mL) was 4.8 months (95% CI 3.81 − 5.79 months) compared to 5.93 months (95% CI 3.05 − 8.19 months) in patients with negative CTM_3–4_ level (*P* = 0.056). Significantly, CTM_3–4_-positive patients show shorter median OS (mOS) (11.13 months, 95% CI 3.52 − 18.74 months) than CTM_3–4_-negative patients (20.43 months, 95% CI 15.80 − 25.06 months, *P* = 0.005) (Fig. 3E). Taken together, although CTM_*2*_ is the dominant subtype in GC-CTM, CTM_3–4_ is the specific subtype that is involved in therapeutic resistance and correlates with prognosis.

### Intra-therapeutic CTM_≥5_ is specifically detected in HER2^+^ GC patients

The larger aggregated CTM_≥5_, which is considered to be more aggressive in tumor metastasis [23, 24], failed to correlate with GC resistance and prognosis in our study. However, as shown in Table 2, all four patients with detectable intra-therapeutic CTM_≥5_ were HER2 positive, and three also had liver metastasis. The obtained results suggest that the formation of CTM_≥5_ might be more influential in driving specific metastatic patterns in GC. HER2^+^ GC with an elevated CTM_≥5_ may be prone to liver metastasis.

**Table 2:** Clinicopathological characteristics of patients with intra-therapeutically positive CTM_≥5_

Patient ID	Primary tumor site	Live metastasis	Lauren classification	HER2 status
P13	Non-GEJ	No	Intestinal	Positive
P16	Non-GEJ	Yes	Intestinal	Positive
P20	GEJ	Yes	Unknown	Positive
P29	Non-GEJ	Yes	Intestinal	Positive

## Discussion

Extending beyond previous demonstrations of the reverse relevance between pre-therapeutic CTM and prognosis [16, 17, 25], this study further concentrates on the clinical significance of dynamic variations in GC-CTM and their heterogeneous size following chemotherapy and targeted therapies. The results indicated that CTM, which was pre-therapeutically detected in 33.3% GC patients, can be further formed in another 34.78% of patients following chemo-/targeted therapies. Positive pre-therapeutic CTM (≥ 1 CTMs/6 mL) correlates with the development of liver metastasis, while the dynamic formation of CTM is involved in therapeutic resistance. Further insight into the size heterogeneity of CTM demonstrated that CTM with distinct cluster sizes could differently contribute to the therapeutic resistance and prognosis of GC. CTM_2_ is the dominant subtype in GC-CTM, while CTM_3–4_ is the specific subtype that is significantly associated with chemo-/targeted therapeutic resistance and inferior PFS and OS. The larger CTM_≥5_, which though failed to show a correlation with prognosis in our study, was more positive in HER2^+^ GC with liver metastasis, implying its crucial role in driving liver colonization of HER2^+^ gastric tumor cells.

Although the underlying biological mechanisms in CTM formation and their role in malignancy have been systematically studied [26], what contributes to the heterogeneous aggregated sizes of the CTM has seldom been addressed. Recently, taking advantage of the developed biophysical model that can mimic cell invasion in vitro, Bocci et al*.* found that distinct epithelial/mesenchymal (E/M) states of the cells in CTM might contribute to the size-heterogenous aggregation of CTM. Hybrid E/M cells are required to organize CTM with 5 − 10 cells, while multiple intermediate E/M states give rise to larger and heterogeneous CTMs formed by cells with different epithelial-mesenchymal traits [9]. Our study supports that the size-heterogeneous aggregation of CTM could further impact the development of therapeutic resistance and metastasis. Medium-sized CTMs, such as CTM_3–4_, are specific components that could drive chemo-/targeted therapeutic resistance in GC, while those aggregated by ≥ 5 cells are more likely to be involved in HER2-driven liver metastasis. Further studies should shed light on how the physical or biological distinctions in GC-CTM_3–4_ and GC-CTM_≥5_ fuel specific phases in cancer development.

In addition, the results obtained in this study also suggest that specific size-based profiling of GC-CTM should be emphasized beyond whole CTM enumeration in terms of clinical resistance or metastasis monitoring. Moreover, quantitative variations of size-heterogeneous CTM should be longitudinally evaluated instead of just pre-therapeutic detection, since the observed formation of CTM following chemo-/targeted therapies. In particular, larger GC-CTM_≥5_ was only observed to form intra-therapeutically, which is in line with recent discoveries based on patient-derived xenograft models that clustered tumor cells resulting from the aggregation of individual CTCs following migration and circulation rather than cohesive shedding [27]. These results make real-time monitoring of larger aggregated CTM following cancer development more meaningful in surveilling metastasis.

In conclusion, our study demonstrates the size heterogeneity of GC-CTM and its involvement in the development of chemo-/targeted therapeutic resistance and metastasis. The results showed that GC-CTM_3–4_ is a potential indicator of therapeutic resistance, while the dynamic presence of GC-CTM_≥5_ implies liver metastasis in HER2^+^ GC patients. Current discoveries highlight the clinical significance of GC-CTM size profiling and their longitudinal monitoring in therapeutic resistance and metastasis surveillance, although further studies with larger sample sizes are needed to validate particularly larger CTM_≥5_ and their correlation with distant metastases.
